# Evaluation of medical student self-rated preparedness to care for limited english proficiency patients

**DOI:** 10.1186/1472-6920-11-26

**Published:** 2011-06-01

**Authors:** Fatima Rodriguez, Amy Cohen, Joseph R Betancourt, Alexander R Green

**Affiliations:** 1 Harvard Medical School, 25 Shattuck Street, Boston, MA, USA; 2 Harvard School of Public Health, 677 Huntington Ave, Boston, MA, USA; 3 Mongan Institute of Health Policy, Massachusetts General Hospital, 50 Staniford Street, Boston, MA, USA; 4 The Disparities Solutions Center, 50 Staniford Street, Massachusetts General Hospital, Boston, MA, USA

## Abstract

**Background:**

Patients with limited English proficiency (LEP) represent a growing proportion of the US population and are at risk of receiving suboptimal care due to difficulty communicating with healthcare providers who do not speak their language. Medical school curricula are required to prepare students to care for all patients, including those with LEP, but little is known about how well they achieve this goal. We used data from a survey of medical students' cross-cultural preparedness, skills, and training to specifically explore their self-rated preparedness to care for LEP patients.

**Methods:**

We electronically surveyed students at one northeastern US medical school. We used bivariate analyses to identify factors associated with student self-rated preparedness to care for LEP patients including gender, training year, first language, race/ethnicity, percent LEP and minority patients seen, and skill with interpreters. We used multivariate logistic regression to examine the independent effect of each factor on LEP preparedness. In a secondary analysis, we explored the association between year in medical school and self-perceived skill level in working with an interpreter.

**Results:**

Of 651 students, 416 completed questionnaires (63.9% response rate). Twenty percent of medical students reported being very well or well-prepared to care for LEP patients. Of these, 40% were in their fourth year of training. Skill level working with interpreters, prevalence of LEP patients seen, and training year were correlated (p < 0.001) with LEP preparedness. Using multivariate logistic regression, only student race/ethnicity and self-rated skill with interpreters remained statistically significant. Students in third and fourth years were more likely to feel skilled with interpreters (p < 0.001).

**Conclusions:**

Increasingly, medical students will need to be prepared to care for LEP patients. Our study supports two strategies to improve student preparedness: training students to work effectively with interpreters and increasing student diversity to better reflect the changing US demographics.

## Background

The United States is becoming more diverse, with increases among racial and ethnic minorities and immigrants, many of whom have difficulty communicating in English. According to recent estimates, over 55 million Americans, or 20% of the total population, speak a language other than English at home [1]. Of these individuals, over half self-report speaking English less than "very well" and are considered to have Limited English Proficiency (LEP) [2]. Medical student education will need to respond to this growing demand by strengthening student skills in working with an increasingly ethnically and linguistically diverse patient population [3,4].

Limited English proficiency is independently associated with adverse health outcomes and preventable medical errors [2,5,6], reduced access to health services [7], longer hospital stays [8], decreased satisfaction with medical care [9], and impaired patient comprehension of medical problems and treatment [10]. The provision of trained medical interpreter services has been shown to significantly improve the quality of care and satisfaction with interpersonal aspects of care for LEP patients [2,4,11,12]. Reinforcing the rights of the patient, clarification of Title VI of the 1964 Civil Rights Act in 2000, mandated that healthcare providers who receive federal funds must provide access to professional language services to any LEP patient, free of cost to the patient [13].

Despite these facts, physicians underuse and misuse interpreters and rely on their own language skills or untrained hospital staff, family, or friends [14]. Recent attention has focused on the role of medical education in improving care for diverse populations [15]. Over the last several years, accrediting bodies such as the Liaison Committee on Medical Education (LCME) and the Accreditation Council for Graduate Medical Education (ACGME) have developed cultural competency training standards and requirements [16-18]. Integral to this teaching is an emphasis on language barriers as a source of health disparities and how to overcome these barriers through skillful utilization of interpreter services [4,19]. However, little is known about how well medical schools prepare their students to care for LEP patients.

Using a previously validated survey tool [20-22], we sought to explore medical student preparedness to care for LEP patients across their four years of training, and to identify factors that might predict this preparedness.

## Methods

### Survey Design and Administration

We analyzed data from a survey entitled, "Medical Students Preparedness to Deliver Cross-cultural Care and Perspectives on Cross-cultural Training at HMS," developed by Green et al [23]. The majority of the survey questions were derived from a previously validated national survey used to explore preparedness to provide cross-cultural care among physicians completing their residency training [20]. The questions were modified and some were added to be more applicable to medical students' experiences. The survey explores three domains of cross-cultural care: 1) attitudes; 2) preparedness; and 3) training, as well as basic personal and professional characteristics.

The survey was administered electronically from August to October 2009 to all medical students, years one through four, at Harvard Medical School. This corresponds to the beginning of the academic year. Dental and MD/PhD students were excluded from the study due to differences in training schedule. Participation in the online survey was voluntary for all students. The Institutional Review Boards of both Harvard Medical School and Partners HealthCare System approved the research protocol.

### Variables

#### Response Variable

To assess medical student self-perceived preparedness to care for LEP patients, we asked how prepared they believed they were to care for LEP patients on a 5-point scale (1 = "very unprepared"; 2 = "somewhat unprepared"; 3 = "somewhat prepared"; 4 = "well-prepared"; and 5 = "very well-prepared").

#### Explanatory Variables

We explored the relationship between medical student preparedness to care for LEP patients and self-reported socio-demographic characteristics of gender, race/ethnicity (White non-Hispanic, Black non-Hispanic, Asian or Pacific Islander, Native American or Alaskan Native, Hispanic/Latino, or other), and first language (English versus other).

We then examined the effects of year of medical school training, prevalence of LEP patients seen, prevalence of minority patients seen, and self-reported skill level in effectively working with an interpreter, on students' level of preparedness caring for LEP patients.

In a secondary analysis, we compared self-perceived skill at working with an interpreter by year in medical school.

### Analysis

We used the χ^2 ^statistic for all bivariate analyses. We then used multivariate logistic regression to determine the relationship between explanatory variables and LEP preparedness. We did not include percent LEP or percent minority patients seen in the model because these questions were not answered by a large proportion of our sample. We included Hispanic/Latino/a, Asian or Pacific Islander, Black non-Hispanic, White non-Hispanic, and Other as the five race/ethnic groups for our model. Native Americans and Pacific Islanders (n = 3) were combined with the "Other" category for our multivariate model. In a sub-analysis, we collapsed medical student race/ethnicity into Hispanic vs. others to increase the power for a small group (n = 21). A total of 368 observations were included in the model.

For analytic purposes, we dichotomized our response variable, LEP preparedness into two groups "prepared" (responses 3,4,5) compared with "unprepared" (responses 1,2). For ease of comparison in our logistic regression model, the 5-point scale for skill level with interpreters was collapsed into three categories: "skilled" (responses 4,5), "intermediate" (response 3), and "not skilled" (responses 1,2).

All analyses were performed using JMP software version 9 (SAS, Inc.). All p-values are two-tailed and a value of <0.05 was considered statistically significant.

## Results

Of 651 students, 444 completed questionnaires and 28 were excluded for missing data, leaving 416 total respondents (response rate 63.9%). Twenty percent of all surveyed medical students reported being very well or well-prepared to care for limited English Proficiency patients. Of these students, 40% were in their fourth year of medical school. For ease of comparison, students who were not included in our final model due to incomplete data were excluded from our unadjusted analysis. Students in year 3 or 4 were more likely to feel prepared but no differences were noted for gender, race/ethnicity, or first language for those who were prepared compared to those who were unprepared (Table 1).

**Table 1:** Characteristics of Study Sample

	All Students	LEP preparedness							
	
		Prepared				Unprepared			P value
	
	N = 368	N		%		N		%	
**Gender**									**0.55**

Male	44.8%	90		43.5%		75		46.6%	

Female	55.2%	117		56.5%		86		53.4%	

**First language**									0.58

English	75.0%	153		73.9%		123		76.4%	

Other	25.0%	54		26.1%		38		23.6%	

**Year in medical school**									<0.001*

Year 1	27.7%	43		20.8%		59		36.6%	

Year 2	24.2%	40		19.3%		49		30.4%	

Year 3	18.2%	45		21.7%		22		13.7%	

Year 4	22.8%	61		29.5%		23		14.3%	

Other	7.1%	18		8.7%		8		5.0%	

**Race/Ethnicity**									**0.11**

White, not Hispanic	46.7%	106		51.2%		66		41.0%	

Black, not Hispanic	6.8%	11		5.3%		14		8.7%	

Asian or Pacific Islander	31.3%	55		26.6%		60		37.3%	

Native American or Alaskan	0.8%	2		1.0%		1		0.6%	

Hispanic/Latino	5.7%	15		7.2%		6		3.7%	

Other	8.7%	18		8.7%		14		8.7%	

*P < 0.05, as significant.

**Figure 1:**
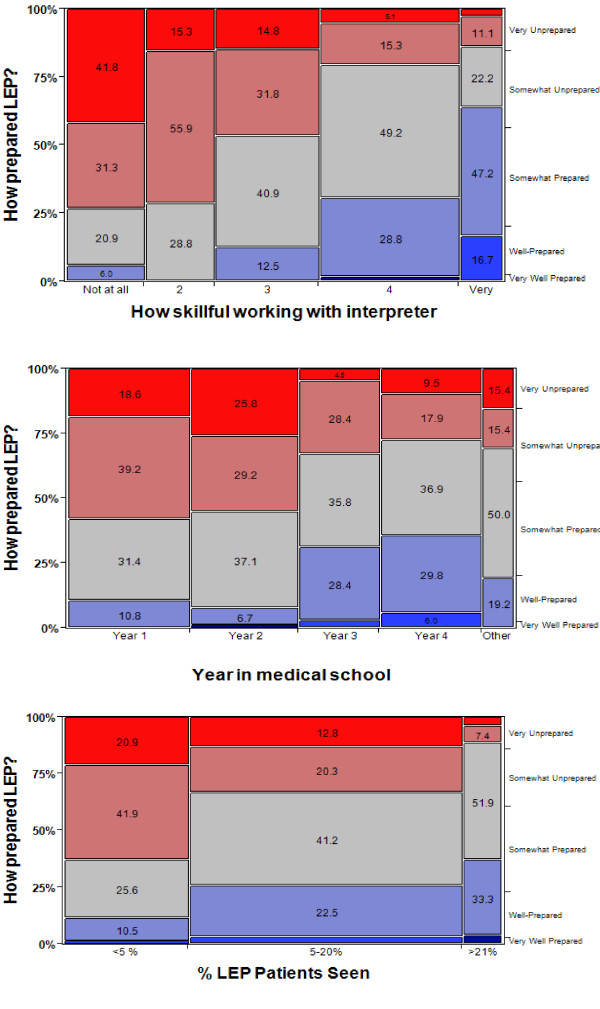
**Mosaic Plots**. a. Contingency Analysis of How Prepared to Care for LEP by How Skillful Working with Interpreter χ^2 ^= 162.779, p < 0.001* b. Contingency Analysis of How Prepared to Care for LEP by Year in Medical School χ^2 ^= 66.216, p < 0.001* c. Contingency Analysis of How prepared to care for LEP by Prevalence of LEP patients χ^2 ^= 51.269, p < 0.001*

χ^2 ^analyses of the ordinal data before dichotomization showed that self-reported skill level with interpreters (p < 0.001), year in medical school (p < 0.001), race/ethnicity (p = 0.028), prevalence of minority patients (p = 0.034), and prevalence of LEP patients (p < 0.001) were significantly correlated with perceived preparedness to care for LEP patients (Figure 1). Specifically, Hispanic medical students in years 3 or 4 who saw a higher proportion of minority and LEP patients felt the most prepared to care for LEP patients.

A total of 368 complete observations were included in the multivariate logistic regression model. In a sensitivity analysis, there was no difference in outcomes for those who were excluded for missing data and those who answered all of the study variables (p = 0.3). In this model, race/ethnicity and self-perceived skill level working with interpreters were significantly associated with preparedness to care for LEP patients, p = 0.03 and p < 0.001, respectively (Table 2). Adjusting for other factors, students who felt skilled working with interpreters had 10 times the odds of feeling prepared to work with LEP patients (95% CI (5.24, 21.4)) compared to those who felt less skilled. Hispanic ethnicity was independently associated with a 2 to 5-fold increase in the odds of feeling prepared to care for LEP patients as compared to White non-Hispanics, Asians or Pacific Islanders, Black non-Hispanics, and other race/ethnic categories, with all comparisons being statistically significant except for Whites. In sub-analysis comparing Hispanics to all others, Hispanics had a 3-fold increase in the odds of being prepared to care for LEP patients (Table 2). Gender, year in medical school, and first language were not statistically associated with self-reported preparedness in the multivariate logistic regression model.

**Table 2:** Factors Associated with Medical Student Preparedness to Care for LEP Patients - Multivariate Logistic Regression (N = 368)

Characteristic	Odds Ratio	Confidence Interval	P-Value
**Gender 0.19**			

Male	(Reference)		

Female	1.3	(0.80, 2.13)	0.19

**Skill level with interpreter**			<0.001*

Not Skillful (1,2)	(Reference)		

Intermediate (3)	2.95	(1.59, 5.57)	<0.001*

Skillful (4,5)	10.76	(5.40, 22.30)	<0.001*

**Year in Medical School**			**0.93**

Year 1	(Reference)		

Year 2	1.22	(0.44, 1.65)	0.65

Year 3	0.81	(0.36, 1.79)	0.49

Year 4	1.13	(0.52, 2.42)	0.47

Other	0.92	(0.31, 2.79)	0.88

**Race/Ethnicity**			0.03*

Hispanic/Latino/a	(Reference)		

White, not Hispanic	0.49	(0.15, 1.48)	0.21

Asian or Pacific Islander	0.27	(0.08, 0.80)	0.02*

Black, not Hispanic	0.22	(0.05, 0.89)	0.03*

Other	0.23	(0.06, 0.08)	0.03*

Hispanic/Latino/a	**3.13**	(1.02, 9.72)	0.047*

Other	(Reference)		

**First Language**			**0.13**

English	(Reference)		

Other	1.63	(0.87,3.08)	0.13

**Figure 2:**
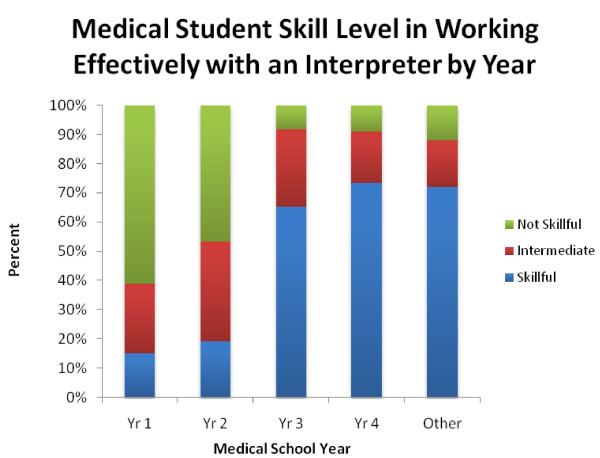
**Medical Student Skill Level in Working Effectively with an Interpreter by Year**. χ^2 ^= 138.87, p < 0.001*

Since self-perceived skill level in working effectively with interpreters appeared to be the strongest predictor of preparedness to work with LEP patients, we then compared self-reported skill level working with interpreters across medical school years. As medical school year advanced, students were increasingly likely to report feeling skillful in working with medical interpreters, with the largest increase occurring between years 2 and 3 (Figure 2).

## Discussion

An influential report by the Institute of Medicine, *Unequal Treatment*, identified several strategies to reduce the growing problem of racial and ethnic disparities in healthcare [24]. Among these, the authors recommended that healthcare professionals should receive training in cultural competence and cross-cultural care and that efforts should be made to recruit a diverse healthcare workforce. Our study's findings underscore these recommendations. We found that the strongest independent predictors of medical student preparedness to work with limited English proficiency patients were students' self-perceived skill level in working with interpreters and students' race/ethnicity.

Teaching specific cross-cultural communication skills to medical students and residents, such as how to effectively work with interpreters, is one practical strategy to address healthcare disparities faced by LEP patients. In our study, we found that the greatest increase in preparedness to care for LEP patients occurred between the beginning of second-year and the beginning of third-year of medical school. While the main purpose of this study was not to evaluate a specific intervention, it should be noted that early in the second year at Harvard Medical School, as part of the longitudinal Patient-Doctor II course, medical students participate in a session devoted to working effectively with interpreters. This session is held at each of the Harvard affiliated hospital sites. In general, the session consists of a presentation by a representative from the hospital's interpreter services department followed by the opportunity to observe an interview between an interpreter, LEP patient, and a healthcare provider. Depending on the availability of LEP patients at a given hospital site, some students are able to briefly practice working with an interpreter as part of this session. To our knowledge this is the only formal interpreter training experience offered at our institution, and may explain some of the increased level of preparedness observed for second year students. Medical students may have additional *ad hoc *opportunities to work with interpreters during their clinical rotations and extracurricular activities, although we are unable to gather this information from our survey instrument.

While preparedness continued to increase between third and fourth year students, it did not appear to be independently related to the percentage of LEP patients students encountered. Instead, it is most likely explained by the 'skill with interpreters' variable, which may be the most important step in a causal pathway. Our data suggest that training of medical students on effectively working with interpreters may lead to better preparedness to care for LEP patients.

Resident physicians have been shown to underuse interpreters, even when readily available [14]. A national survey of resident physicians also showed that physicians who received training in the area of cross-cultural care felt more prepared to care for a diverse patient population [21]. As the Joint Commission and other federal and state agencies establish stricter guidelines on interpretation, it will be critical to prepare future physicians to understand the impact of language barriers and how to effectively use interpreter services to help them meet these standards [25]. It is likely that training of this type would remove some of the barriers associated with interpreter underuse and misuse in residency and in future clinical practice.

Our finding that Hispanic medical students feel more prepared to care for LEP patients supports the notion that an important approach to reduce racial/ethnic health disparities is to increase the diversity of the healthcare workforce [24]. Research has shown that minority patients experience higher levels of satisfaction with their medical care when cared for by racially and ethnically concordant physicians [26-28]. Similarly, LEP patients report greater satisfaction with interpersonal aspects of care and improved clinical outcomes with concordant language providers [29-31]. Given that Hispanics are the largest and fastest growing segment of the US population - many of whom are LEP - these findings support that medical schools' recruitment efforts should target Hispanic students to better represent this growing ethnic and linguistic diversity.

Surprisingly, medical students' first language was not statistically associated with increased preparedness to care for LEP patients. We hypothesize that this may be partially attributed to the fact that Harvard affiliated hospitals serve a largely Spanish-speaking LEP population. Many of our students whose first language is not English likely do not speak Spanish. As is shown in Table 1, over 31% of the surveyed student body are Asian or Pacific Islander and only 6% are Hispanic or Latino. However, we did not explicitly inquire about students' first language other than English or second language fluency, which limits our ability to better interpret the finding. Furthermore, it is also possible that many Hispanic students speak Spanish as a second language and/or that using an interpreter effectively allowed primary English speakers to feel equally comfortable caring for LEP patients.

The effect of medical school year was also lost in our multivariate analysis, most likely because it was a marker of a more powerful predictor - increased skill level at effectively working with an interpreter.

Our study has several limitations. The response rate of 64% may introduce bias if non-responders were systematically different from responders. However, this rate compares favorably with that of similar studies [20]. The study's cross-sectional design limits our ability to make inferences about causality. Because this survey was conducted at one medical school in the Northeastern US, the results may not be generalizable to all medical schools. Our data is self-reported and may not actually represent what medical students do in practice. A recent study by Thompson et al. suggests that medical students tend to overestimate their cultural competency/skill level [32]. However, in our study we found that medical students rated their preparedness to care for LEP patients lower than other variables of cross-cultural preparedness. The survey did not ask about students' ability to speak a second language, which may have correlated with preparedness to care of LEP patients. Future studies should longitudinally track and measure medical student skills to provide cross-cultural care. These skills should also be measured and evaluated in systematic ways through OSCE examinations and other methods [4,33]. Ultimately, studies should explore how interventions in cross-cultural medical education translate into improved patient care and outcomes for LEP populations [19,34].

## Conclusions

Improving physician skills in cross-cultural care is an important strategy to address health disparities for an increasingly diverse patient population. Undergraduate medical education provides a critical target for intervention. Our study found that two factors independently predicted self-rated preparedness to care for LEP patients: self-perceived skill level at working effectively with an interpreter and Hispanic ethnicity. Further research is warranted to directly evaluate the impact of controlled educational interventions as well as the effect of diversifying the physician workforce on improving the care of LEP patients.

## Competing interests

The authors declare that they have no competing interests.

## Authors' contributions

FR carried out the study design, analysis, and writing of the manuscript. AC contributed to the statistical analysis and review of the manuscript. JRB contributed to the review and editing of the manuscript. ARG conceived the study design and contributed to data collection, analysis, and writing of the manuscript. All authors have reviewed and approved the final version of the manuscript.

## Authors' information

FR is a fourth year medical student at Harvard Medical School and an MPH candidate at the Harvard School of Public Health. AC is the Instructional Computing Manager at the Harvard School of Public Health. JRB is the Director of the Disparities Solutions Center at Massachusetts General Hospital and an Associate Professor of Medicine at Harvard Medical School. ARG is the Associate Director of the Disparities Solutions Center at Massachusetts General Hospital and Chair of the Cross-Cultural Care Committee at Harvard Medical School.

## Pre-publication history

The pre-publication history for this paper can be accessed here:

http://www.biomedcentral.com/1472-6920/11/26/prepub
